# Central regulation of brown adipose tissue thermogenesis and energy homeostasis dependent on food availability

**DOI:** 10.1007/s00424-017-2090-z

**Published:** 2017-12-05

**Authors:** Yoshiko Nakamura, Kazuhiro Nakamura

**Affiliations:** 1 Department of Integrative Physiology, Nagoya University Graduate School of Medicine, Nagoya, 466-8550 Japan; 2 PRESTO, Japan Science and Technology Agency, Kawaguchi, Saitama 332-0012 Japan

**Keywords:** Brown adipose tissue, Feeding, Hunger, Metabolism, Sympathetic, Thermoregulation

## Abstract

Energy homeostasis of mammals is maintained by balancing energy expenditure within the body and energy intake through feeding. Several lines of evidence indicate that brown adipose tissue (BAT), a sympathetically activated thermogenic organ, turns excess energy into heat to maintain the energy balance in rodents and humans, in addition to its thermoregulatory role for the defense of body core temperature in cold environments. Elucidating the central circuit mechanism controlling BAT thermogenesis dependent on nutritional conditions and food availability in relation to energy homeostasis is essential to understand the etiology of symptoms caused by energy imbalance, such as obesity. The central thermogenic command outflow to BAT descends through an excitatory neural pathway mediated by hypothalamic, medullary and spinal sites. This sympathoexcitatory thermogenic drive is controlled by tonic GABAergic inhibitory signaling from the thermoregulatory center in the preoptic area, whose tone is altered by body core and cutaneous thermosensory inputs. This circuit controlling BAT thermogenesis for cold defense also functions for the development of fever and psychological stress-induced hyperthermia, indicating its important role in the defense from a variety of environmental stressors. When food is unavailable, hunger-driven neural signaling from the hypothalamus activates GABAergic neurons in the medullary reticular formation, which then block the sympathoexcitatory thermogenic outflow to BAT to reduce energy expenditure and simultaneously command the masticatory motor system to promote food intake—effectively commanding responses to survive starvation. This article reviews the central mechanism controlling BAT thermogenesis in relation to the regulation of energy and thermal homeostasis dependent on food availability.

## Introduction

Mammals including humans maintain energy homeostasis by balancing energy intake and expenditure. A major physiological activity that expends energy within the body of these homeothermic animals is adaptive heat production (thermogenesis), which is particularly essential to maintain body core temperature under subthermoneutral environmental temperature. Adaptive thermogenesis in brown adipose tissue (BAT) is well known as a major sympathetic response for cold defense in rodents [7]. BAT thermogenesis is also known to occur for cold defense in adult humans, in which the thermogenic capacity in BAT has been shown to inversely correlate with obesity indices and with fat mass of subjects [13, 78, 96]. Therefore, BAT seems to function to turn excess energy into heat under well-fed conditions to maintain the homeostatic energy balance, in addition to the defense of thermal homeostasis in cold environments.

Under strong hunger, on the other hand, adaptive thermogenesis in BAT is suppressed to save energy even in cold environments, often resulting in hypothermia [79, 103, 107]. The regulation of thermogenesis dependent on food availability and nutritional conditions is essential for mammals to maintain energy homeostasis and to survive starvation. Mammals have acquired the mechanism to reduce thermogenesis during hunger through the long history of evolution, during most of which they were faced with hunger. The mechanism that regulates thermogenesis in response to hunger and satiety is located in the brain, and the neural circuit is linked with the circuits controlling food intake so that the brain can effectively control autonomic and behavioral effector organs to defend energy homeostasis under hunger and satiated conditions.

Elucidating the central circuit mechanisms that control energy expenditure (thermogenesis) and food intake for energy homeostasis is important in understanding the etiology of obesity as well as the fundamental mechanism of survival responses to hunger and starvation. In this review article, we describe the current understandings of the basic central circuit mechanisms controlling BAT thermogenesis for cold defense, fever and psychological stress-induced hyperthermia, and then, of the central regulation of BAT thermogenesis and energy homeostasis dependent on food availability, with a special focus on a recently identified circuit that commands responses to hunger and starvation.

## Roles of BAT thermogenesis in energy homeostasis and cold defense

Physiological responses for the autonomous regulation of body core temperature in mammals include heat production within the body and heat loss from the body surface, both of which are controlled by central neural circuits [54]. One of the effector organs controlling heat loss is skin blood vessels, which primarily receive sympathetic innervation that alters skin blood flow to control radiant heat loss. On the other hand, BAT is a major thermogenic organ particularly in rodents. BAT also receives abundant sympathetic innervation and brown adipocytes are stimulated by catecholamines through β_3_-adrenoceptors on their surface [7]. The activation of β_3_-adrenoceptor-mediated intracellular signaling results in heat production by uncoupling protein 1 (UCP1) in mitochondria [7]. Indicating the importance of BAT thermogenesis in energy homeostasis and cold defense, genetic ablation of BAT in mice results in obesity, increased total body lipid and intolerance to cold [35]. Despite its small mass in total body weight, BAT has also been shown as a major organ that clears and combusts circulating triglycerides and takes up glucose particularly when animals are exposed to cold [4].

β_3_-Adrenoceptors are expressed primarily in BAT [53], and chronic systemic infusion of a β_3_-adrenoceptor agonist in rats of high-fat diet-induced obesity increases body core temperature, energy expenditure and UCP1 content in BAT, and reduces the weights of white adipose tissue depots without altering food intake, ameliorating the obesity [20]. β_3_-Adrenoceptor-deficient mice show a mild obesity phenotype with modestly increased fat stores [89], and mice lacking the three known β-adrenoceptor subtypes are more obese on a standard chow diet and even more severe on a high-fat diet, show lower basal metabolic rate, and lack cold-induced thermogenesis and UCP1 increase in BAT, leading to hypothermia [2]. These findings from rodent studies indicate the significant contribution of β_3_-adrenoceptor-mediated thermogenesis in BAT to prevent obesity and also suggest the involvement of other adrenoceptor subtypes in BAT thermogenesis for energy homeostasis.

Physiological significance of BAT thermogenesis in adult humans has been demonstrated by studies using positron-emission tomographic and computed tomographic (PET/CT) scanning. This imaging technique can visualize body cooling-induced accumulation of fluorodeoxyglucose (FDG), a glucose analog, in supraclavicular and paraspinal regions, which harbor fat depots rich in adipocytes expressing UCP1, a marker of brown adipocytes [13, 78, 96, 97]. Therefore, the cooling-induced FDG accumulation has been considered indicative of BAT thermogenesis in human subjects. Of note, cooling-induced FDG accumulation, if found, is generally higher in winter than summer, and the interindividual variation in the FDG accumulation inversely correlates with body mass index and with fat mass of the subjects [13, 78, 96]. These findings indicate important physiological roles of BAT in prevention of obesity as well as cold defense in adult humans.

## Central circuit mechanism controlling BAT thermogenesis

**Fig. 1:**
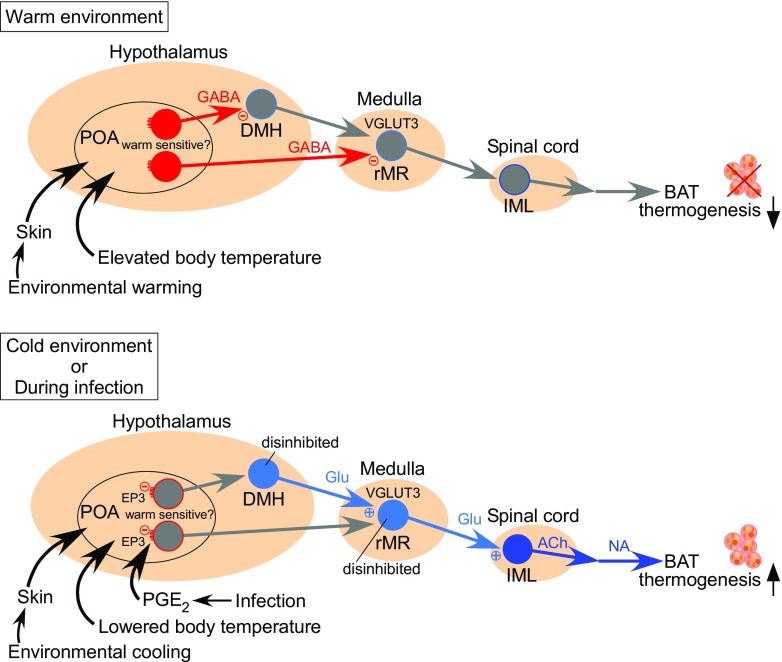
Model of neural circuit controlling BAT thermogenesis for body temperature regulation and fever. In warm environments, an elevation of body core temperature, which is sensed by warm-sensitive neurons in the POA, or cutaneous warm-sensory inputs to the POA lead to activation of GABAergic projection neurons through the local circuit mechanisms in the POA (see the main text). The activated GABAergic neurons projecting from the POA inhibit neurons in the DMH and rMR to suppress BAT thermogenesis. In cold environments, a decrease in body core temperature or cutaneous cool-sensory inputs to the POA lead to inhibition of the GABAergic projection neurons. An action of PGE_2_ on EP3 receptors likely expressed in the GABAergic projection neurons also inhibits the activity of these neurons. The attenuation of the tonic GABAergic inhibition from the POA leads to disinhibition of sympathoexcitatory pathway to drive BAT thermogenesis. For detail, see the main text

**Fig. 2:**
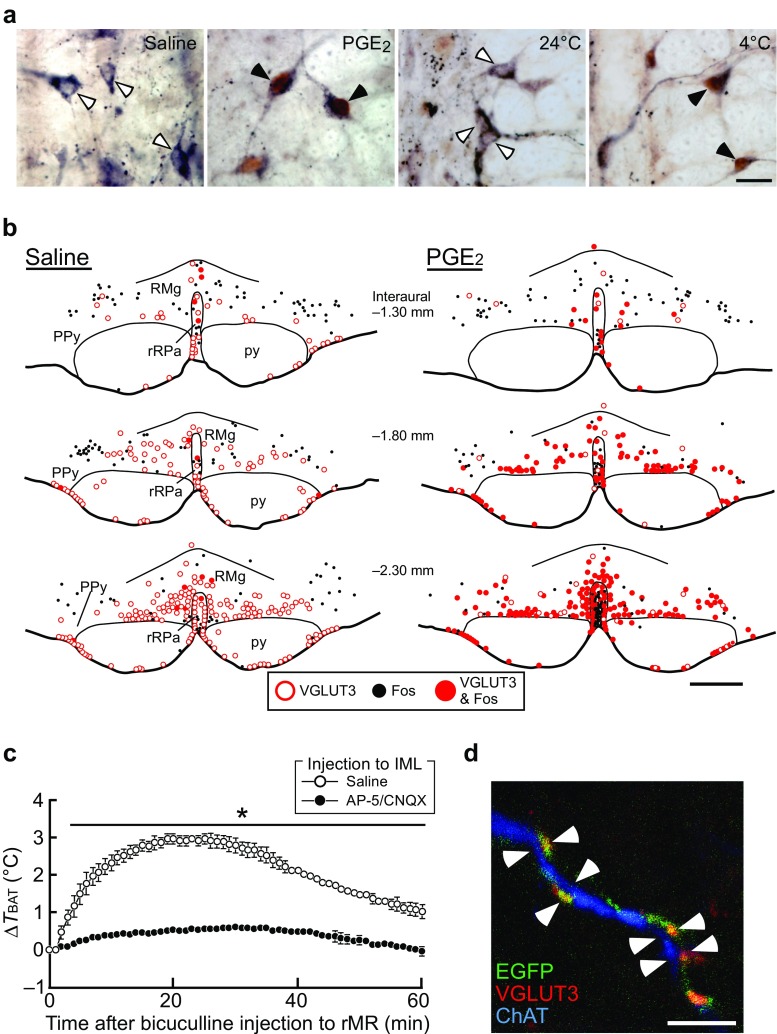
Sympathetic premotor neurons in the rMR that drive BAT thermogenesis in response to thermogenic stimuli. **a** Expression of Fos (brown), a marker for neuronal activation, in VGLUT3-immunoreactive (blue-black) neurons in the rat rMR in response to intracerebroventricular injection of saline or PGE_2_ or exposure of the animals to 24 °C (room temperature) or 4 °C (cold). Open and filled arrowheads indicate VGLUT3-immunoreactive neuronal cell bodies that are negative and positive for Fos immunoreactivity, respectively. Scale bar, 20 μm. **b** Distribution of VGLUT3- and Fos-immunoreactive neurons in the rat rostroventral medulla. PPy, parapyramidal region; py, pyramidal tract; RMg, raphe magnus nucleus; rRPa, rostral raphe pallidus nucleus. Scale bar, 500 μm. **c** Nanoinjections of glutamate receptor antagonists (AP-5/CNQX) into the IML block BAT thermogenesis induced by stimulation of sympathetic premotor neurons in the rMR with a nanoinjection of bicuculline, a GABA_A_ receptor antagonist. Temperature changes in the rat interscapular BAT (*T*_BAT_) after a bicuculline injection into the rMR are compared between the groups that had prior injections of AP-5/CNQX (*n* = 4) or saline (*n* = 3) into the IML over the T2–T6 spinal segments. All values are means ± SEM. *P < 0.05. **d** A confocal image in the IML showing that rMR-derived axon fibers containing both enhanced green fluorescent protein (EGFP) and VGLUT3 are closely associated with dendritic fibers of sympathetic preganglionic neurons immunoreactive for choline acetyltransferase (ChAT). Scale bar, 5 μm. Modified from Nakamura et al. [57] with permission

Figure 1 shows the current model of the basic central neural circuit mechanism controlling BAT thermogenesis for thermoregulation and fever [54]. The sympathetic preganglionic neurons that directly control the postganglionic neurons innervating BAT are localized in the intermediolateral cell nucleus (IML) of the spinal cord. These preganglionic neurons are innervated by sympathetic premotor neurons that are distributed in the rostral medullary raphe region (rMR) consisting of the rostral raphe pallidus and raphe magnus nuclei [57, 59]. These BAT sympathetic premotor neurons express vesicular glutamate transporter 3 (VGLUT3), a putative marker of glutamatergic neurons [57] (Fig. 2). Predominant populations of VGLUT3-expressing neurons in the rMR innervate BAT and skin blood vessels through their projecting axons synapsing on sympathetic preganglionic neurons in the spinal cord [57, 65, 88] (Fig. 2d), indicating that VGLUT3-expressing neurons in the rMR are sympathetic premotor neurons controlling thermoregulatory effectors. Many of VGLUT3-expressing neurons in the rMR are activated in response to physiological and pathological thermogenic stimuli given to animals, such as cold exposure, central injection of a pyrogenic mediator, and psychological stress [34, 57] (Fig. 2a, b). Suppression of activity of neuronal cell bodies in the rMR with local nanoinjections of muscimol, a GABA_A_ receptor agonist widely used as a neuronal inhibitor, completely blocks the induction of BAT thermogenesis by cold exposure, central injection of a pyrogenic mediator, and psychological stress [26, 50, 58, 60, 64]. On the other hand, stimulation of neurons in the rMR induces BAT thermogenesis [38, 49, 52, 73] and this thermogenic response is blocked by injections of glutamate receptor antagonists into the IML of the spinal cord [57] (Fig. 2c). These findings support the view that activation of the rMR-spinal glutamatergic sympathetic premotor transmission is an essential step in the central signaling outflow to drive BAT thermogenesis.

In addition to the principal transmitter role of glutamate in the rMR-spinal thermogenic drive, serotonin has been shown to modulate the glutamatergic synaptic transmission in the spinal cord. A small population (10–20%) of VGLUT3-expressing neurons in the rMR contains serotonin and likely co-releases glutamate and serotonin from their axon terminals in the spinal IML [57, 65, 88]. Nanoinjection of glutamate or a glutamate receptor agonist into the spinal IML elicits a rapid thermogenic response in BAT [40, 57]. This thermogenic response to glutamate receptor stimulation in the IML is potentiated by a prior injection of serotonin into the same site, although serotonin injection by itself does not elicit rapid BAT thermogenesis [40]. These findings suggest that serotonin boosts glutamate-evoked subthreshold depolarizations in BAT sympathetic preganglionic neurons to increase their firing probability.

BAT sympathetic premotor neurons in the rMR receive glutamatergic (VGLUT2-positive) excitatory projections from the dorsomedial hypothalamus (DMH) consisting of the dorsomedial hypothalamic nucleus and dorsal hypothalamic area [26]. DMH neurons projecting to the rMR cluster near the boundary between the ventral edge of the dorsal hypothalamic area and the dorsal edge of the dorsomedial hypothalamic nucleus [21, 67, 80]. These rMR-projecting neurons in the DMH are activated by cold exposure, infection, and psychological stress [26, 81, 101]. Mimicking sympathetic physiological responses to cold exposure, infection and psychological stress, selective stimulation of the DMH-rMR monosynaptic pathway with an in vivo optogenetic technique in rats elicits BAT thermogenesis and tachycardic responses, which are both blocked by antagonizing glutamate receptors in the rMR [26]. Inactivation of neurons in the DMH with muscimol nanoinjections completely blocks BAT thermogenesis evoked by cold exposure, central injection of a pyrogenic mediator, and psychological stress [26, 39, 60, 64, 67, 105]. These findings are consistent with the notion that thermogenic command signals activate glutamatergic DMH neurons projecting to the rMR to stimulate the BAT sympathetic premotor drive (Fig. 1).

The DMH-rMR thermogenic pathway is likely under a tonic GABAergic control by the preoptic area (POA), in which the thermoregulatory center is located. Blockade of GABA_A_ receptors in the DMH or transection of descending outputs from the POA elicits robust BAT thermogenesis [8, 10, 77, 106], indicating that the POA provides tonic GABAergic inhibition to the DMH to control the activity level of the excitatory DMH-rMR thermogenic drive to BAT. The currently proposed model of the thermoregulatory central circuit [51, 54] (Fig. 1) explains that the descending inhibitory transmission from the POA is augmented to inhibit BAT thermogenesis under environmental conditions that do not demand heat, such as in a hot environment. On the other hand, sensory signals transmitting a demand for heat to the POA, such as cold-sensory signals and pyrogenic signals (see below), are thought to decrease the descending inhibition from the POA, resulting in disinhibition of the DMH-rMR thermogenic excitatory drive to BAT [51, 54] (Fig. 1). This model has been confirmed by a recent optogenetic experiment: BAT thermogenesis was inhibited by selective stimulation of a predominantly GABAergic transmission to the DMH from a group of POA neurons, which express pituitary adenylate cyclase-activating polypeptide (PACAP) and brain-derived neurotrophic factor (BDNF) and can be activated in response to warm-sensory inputs from the skin [92].

The POA contains many warm-sensitive neurons, whose firing activity is increased in response to an elevation of local tissue temperature [70, 71], and local cooling in the POA elicits BAT thermogenesis [23]. Because brain temperature changes in parallel to temperature changes in other body core structures including visceral organs, the activity of warm-sensitive neurons in the POA likely reflects the level of body core temperature, which is required information for the core temperature-dependent feedback thermoregulatory mechanism [25, 54]. In the feedback mechanism, a warming-induced increase in firing activity of warm-sensitive neurons leads to inhibition of BAT thermogenesis probably through inhibition of DMH neurons (Fig. 1). Consistent with the idea that warm-sensitive POA neurons provide the descending GABAergic inhibition, warm-sensitive neurons identified in primary cultured POA neurons are predominantly GABAergic [91]. However, PACAP/BDNF-expressing neurons in the POA are not warm-sensitive neurons, since they do not show intrinsic thermosensitivity to local warming [92]. Because neither histological nor genetic marker to identify warm-sensitive POA neurons has been available, the sites of their projections and the molecular mechanism of their thermosensitivity remain unknown.

In addition to monitoring brain temperature, the POA also receives information on skin temperature from cutaneous thermoreceptors, which monitor changes in ambient temperature. This feedforward thermosensory signaling from the skin is required for the POA to immediately command “preventive” thermoregulatory responses to changes in ambient temperature before they impact body core temperature [25, 54]. Cutaneous cool-sensory and warm-sensory signals are separately transmitted to the POA through ascending pathways composed of glutamatergic neurons in the spinal dorsal horn and the lateral parabrachial nucleus (LPB) [61, 63] and are likely integrated with the information on brain temperature by impinging on warm-sensitive neurons in the POA [5]. Cutaneous cool-sensory glutamatergic inputs from the LPB to the POA likely inhibit warm-sensitive neuron activity through activation of GABAergic inhibitory interneurons to stimulate BAT thermogenesis and other cold-defensive responses [62, 64], whereas cutaneous warm-sensory glutamatergic inputs to this site could increase warm-sensitive neuron activity through activation of excitatory, potentially glutamatergic, interneurons to inhibit thermogenesis and to increase heat loss [54] (Fig. 1). Consistent with the idea of glutamatergic POA interneurons inhibiting thermogenesis, optogenetic stimulation of glutamatergic neuronal cell bodies in the POA inhibits BAT thermogenesis [84, 104].

The POA is also known as the febrile center, which commands febrile responses including BAT thermogenesis and cutaneous vasoconstriction to increase body temperature during infection or systemic inflammation. Febrile command signaling from the POA is triggered by an action of prostaglandin E_2_ (PGE_2_) on neurons in the POA. PGE_2_, which is biosynthesized in brain endothelial cells in response to immune signaling stimulated by infection [45, 99, 100], acts on prostaglandin EP3 receptors expressed in neurons in the POA [55, 56] to trigger fever [31]. Because the EP3 receptor has been shown in cultured cells as a metabotropic receptor coupled to the inhibitory GTP-binding protein, G_i_ [72], the action of PGE_2_ on POA neurons through EP3 receptors likely inhibits their firing activity. EP3 receptor-expressing POA neurons are predominantly GABAergic and project to the DMH and rMR [58, 67], and furthermore, these POA neurons innervate BAT through multisynaptic neural pathways [102]. These findings support the view (Fig. 1) that EP3 receptor-expressing POA neurons usually control the activity of neurons in the DMH and rMR through their tonic inhibitory transmission for basal thermoregulation and, during infection, an action of PGE_2_ on EP3 receptor-expressing POA neurons to inhibit their tonic activity leads to disinhibition of the DMH and rMR neurons, resulting in stimulated sympathetic outflows to thermoregulatory effectors including BAT to develop fever [54, 58, 67]. However, whether EP3 receptor-expressing POA neurons are warm-sensitive is unknown.

The subpopulations of EP3 receptor-expressing POA neurons projecting to the DMH and to the rMR are distinct [68]. Cooling-induced and febrile BAT thermogenesis requires activation of both DMH and rMR neurons [39, 50, 58, 60, 64, 67, 105], whereas the central efferent pathway for cutaneous vasoconstrictor responses to the same stimuli involves rMR neurons, but bypasses the DMH [77]. Therefore, the subpopulations of EP3 receptor-expressing POA neurons projecting to the DMH and to the rMR might separately regulate BAT thermogenesis and cutaneous vasoconstriction, respectively.

## Neural control of BAT thermogenesis and energy homeostasis during hunger

There are multiple mechanisms for the brain to sense hunger. A well-known humoral mechanism to transmit the information of hunger to the brain is the ghrelin-neuropeptide Y (NPY) pathway. In fasted animals, the stomach releases ghrelin, which is delivered to the hypothalamic arcuate nucleus in the brain through the circulation [28]. Ghrelin activates NPY/agouti-related peptide (AgRP)-containing neurons in the arcuate nucleus [27], which then release NPY from their axonal nerve endings in the paraventricular hypothalamic nucleus (PVH) [24]. The action of NPY on PVH neurons triggers hunger signaling, which stimulates food intake as well as reduces energy expenditure [1, 86, 98]. The hypothalamic NPY-induced reduction of energy expenditure involves inhibition of adaptive BAT thermogenesis that occurs in sub-thermoneutral environments but not reduction of basal metabolic rate [16, 90]. Therefore, the hunger signaling triggered by hypothalamic NPY likely inhibits the sympathetic outflow to BAT by acting on somewhere in the neural pathway controlling adaptive thermogenesis in BAT without affecting basal metabolic activities within the body.

**Fig. 3:**
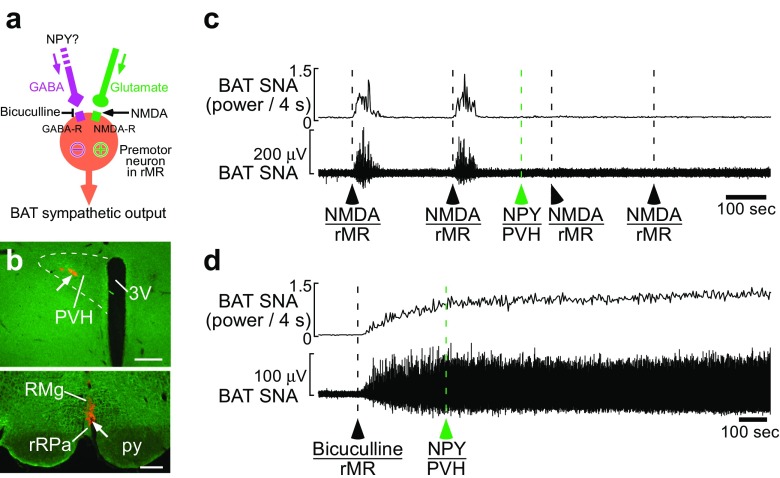
Hypothalamic NPY inhibits BAT thermogenesis through GABAergic transmission to sympathetic premotor neurons. **a** Sympathetic premotor neurons in the rMR are controlled by glutamatergic excitatory and GABAergic inhibitory inputs. **b**–**d** NPY injection into the rat PVH (arrow in (**b**), top) suppressed increases in BAT sympathetic nerve activity (SNA) induced by NMDA nanoinjections into the rMR (arrow in (**b**), bottom) (**c**) but not that induced by blockade of GABA_A_ receptors in the rMR with a bicuculline nanoinjection (**d**). Scale bars, 0.3 mm. 3V, third ventricle. Modified from Nakamura et al. [69] with permission

A recent study demonstrated that the hypothalamic NPY-triggered hunger signaling provides an inhibitory input to BAT sympathetic premotor neurons in the rMR [69] (Fig. 3). Consistent with the view that the BAT sympathetic premotor activity is determined by the balance between excitatory (glutamatergic) inputs and inhibitory (GABAergic) inputs to the premotor neurons [54] (Fig. 3a), a nanoinjection of either glutamate receptor agonist or GABA_A_ receptor antagonist into the rMR evokes BAT thermogenesis [69] (Fig. 3c, d). Nanoinjection of NPY into the rat PVH strongly inhibits BAT thermogenesis induced by skin cooling or that by glutamatergic stimulation of sympathetic premotor neurons in the rMR with a local nanoinjection of NMDA [16, 69] (Fig. 3c). However, the same NPY injection cannot inhibit BAT thermogenesis induced by antagonizing GABA_A_ receptors in the rMR [69] (Fig. 3d). These experimental results indicate that the NPY-triggered hunger signaling from the hypothalamus provides a GABAergic input to BAT sympathetic premotor neurons to overcome excitatory inputs to them, leading to an inhibition of BAT sympathetic premotor drive to the IML. Consistently, VGLUT3-expressing sympathetic premotor neurons in the rMR receive numerous GABAergic synaptic inputs [59]. Although one of the sources of the GABAergic inputs is likely to be EP3 receptor-expressing neurons in the POA [58], the effect of hunger signals on the activity of EP3 receptor-expressing POA neurons has yet to be examined.

**Fig. 4:**
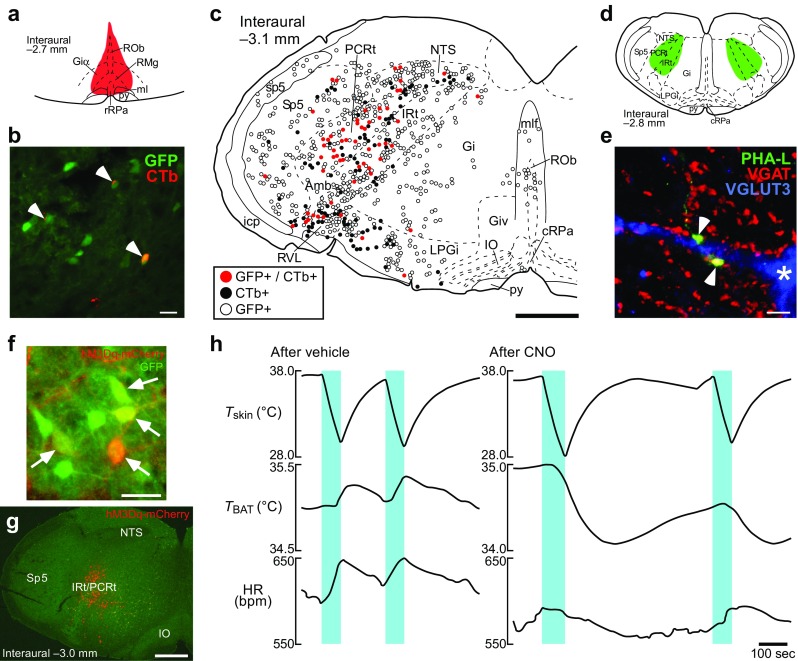
GABAergic neurons in the IRt/PCRt innervate VGLUT3-expressing sympathetic premotor neurons in the rMR to inhibit BAT thermogenesis. **a**–**c** Retrograde neural tracing from the rMR in *Gad1-Gfp* knock-in mice. Injection of the retrograde tracer, cholera toxin b-subunit (CTb) into the rMR ((**a**), red) resulted in labeling of GFP-expressing (representing GABAergic) neurons in the IRt/PCRt with CTb ((**b**), arrowheads). The mapping (**c**) shows the medullary distribution of CTb-labeled and GFP-expressing neurons. Amb, ambiguus nucleus; cRPa, caudal raphe pallidus nucleus; Gi, gigantocellular reticular nucleus; Giα, alpha part of the gigantocellular reticular nucleus; Giv, ventral part of the gigantocellular reticular nucleus; icp, inferior cerebellar peduncle; IO, inferior olivary complex; LPGi, lateral paragigantocellular nucleus; ml, medial lemniscus; mlf, medial longitudinal fasciculus; ROb, raphe obscurus nucleus; RVL, rostral ventrolateral medulla; sp5, spinal trigeminal tract; Sp5, spinal trigeminal nucleus. Scale bars, 30 μm (**b**), 0.5 mm (**c**). **d**, **e** Anterograde tracing from the IRt/PCRt to the rMR in rats. *Phaseolus vulgaris* leucoagglutinin (PHA-L) was injected bilaterally into the IRt/PCRt ((**d**), green). Arrowheads in the pseudocolored confocal image (**e**) indicate apposition of PHA-L-labeled, vesicular GABA transporter (VGAT)-containing (representing GABAergic) axon swellings to dendrites and cell bodies (asterisk) of VGLUT3-immunoreactive neurons in the rMR. Scale bar, 5 μm. **f**–**h** Chemogenetic stimulation of GABAergic IRt/PCRt neurons eliminates cooling-induced BAT thermogenesis and tachycardia. Virus-mediated transduction of GFP-expressing GABAergic neurons in the IRt/PCRt with the G_q_-coupled designer receptor (hM3Dq-mCherry) exclusively activated by designer drug (arrows in (**f**)) in a *Gad2-IRES-Cre*/*Gad1-Gfp* double knock-in mouse (distribution shown in (**g**)). Cooling the skin (*T*_skin_) (blue periods in (**h**)) of the mice induced BAT thermogenesis and tachycardia (heart rate, HR) following bilateral nanoinjections of vehicle into the IRt/PCRt, whereas the sympathetic responses were eliminated after injections of clozapine-*N*-oxide (CNO), a selective agonist for hM3Dq, into the IRt/PCRt. Scale bars, 30 μm (**f**) and 0.5 mm (**g**). Modified from Nakamura et al. [69] with permission

The hypothalamic NPY-driven hunger signaling has recently been shown to provide the inhibition to BAT sympathetic premotor neurons by employing a GABAergic group of neurons in the intermediate (IRt) and parvicellular (PCRt) reticular nuclei of the medulla oblongata [69] (Fig. 4). Neural tract tracing studies in rats and mice revealed that GABAergic neurons in the IRt/PCRt innervate VGLUT3-expressing sympathetic premotor neurons in the rMR [69] (Fig. 4a–e). Stimulation of neurons in the IRt/PCRt inhibits BAT thermogenesis induced by either body cooling or PGE_2_ injection into the POA [69], indicating that IRt/PCRt neurons exert an inhibitory effect on cooling-induced and febrile BAT thermogenesis. However, stimulation of IRt/PCRt neurons, similar to NPY injection into the PVH, cannot inhibit BAT thermogenesis induced by antagonizing GABA_A_ receptors in the rMR [69], consistent with the view that the IRt/PCRt provides GABAergic inhibition to BAT sympathetic premotor neurons in the rMR. Directly demonstrating that GABAergic neurons are responsible for the IRt/PCRt-mediated inhibition of adaptive BAT thermogenesis, selective stimulation of GABAergic neurons in the mouse IRt/PCRt using an in vivo chemogenetic technique suppresses BAT thermogenesis evoked by body cooling [69] (Fig. 4f–h).

**Fig. 5:**
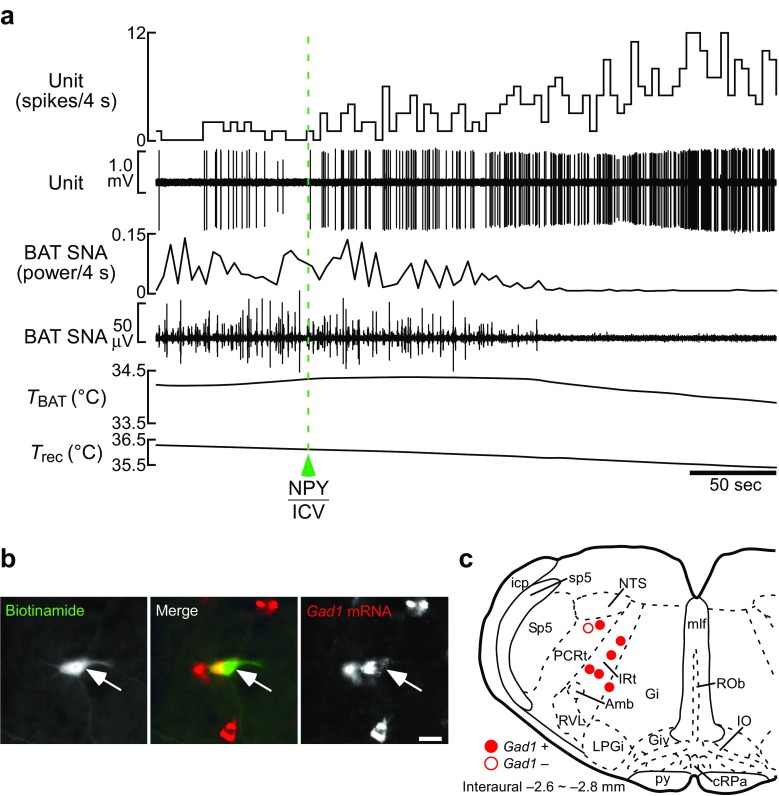
NPY-triggered hunger signaling activates GABAergic IRt/PCRt neurons innervating the rMR. **a** In vivo extracellular unit recording of action potentials of a rat IRt/PCRt neuron, which was confirmed to project to the rMR with a collision test [69]. An intracerebroventricular (ICV) NPY injection increased the firing rate of this neuron (unit) and reversed BAT SNA and *T*_BAT_ that had been increased by moderate body cooling. *T*_rec_, rectal temperature. **b** Histochemical identification of a recorded IRt/PCRt neuron innervating the rMR with juxtacellular labeling (green, arrow). In situ hybridization revealed that this neuron expressed *Gad1* mRNA (red), a marker of GABAergic neurons. Scale bar, 30 μm. **c** Location of rMR-projecting IRt/PCRt neurons successfully identified with juxtacellular labeling. Among them, all neurons activated by NPY injection expressed *Gad1* (filled circles), whereas the *Gad1*-negative one did not respond to NPY injection (open circle). Modified from Nakamura et al. [69] with permission

**Fig. 6:**
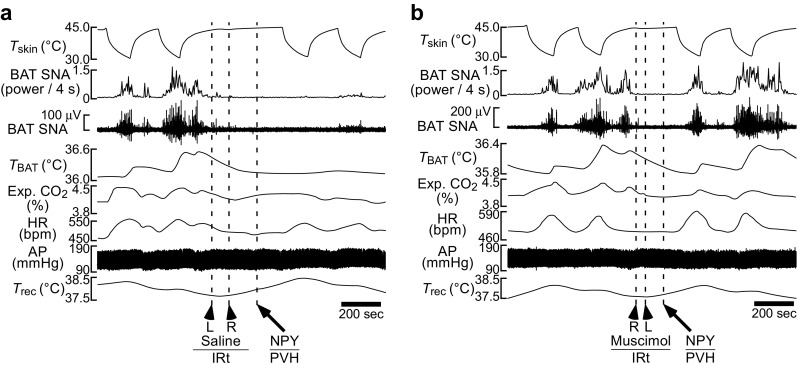
Activation of IRt/PCRt neurons are required for hypothalamic NPY-induced inhibition of BAT thermogenesis, metabolism and tachycardia. **a**, **b** Skin cooling-evoked increases in BAT thermogenesis, whole body metabolism (expired CO_2_) and HR were inhibited by an NPY nanoinjection into the rat PVH following bilateral nanoinjections of saline into the IRt/PCRt (**a**), whereas bilateral nanoinjections of muscimol to inactivate IRt/PCRt neurons prevented the inhibitory effects of NPY (**b**). AP, arterial pressure. Modified from Nakamura et al. [69] with permission

In vivo unit recordings from single IRt/PCRt neurons in rats in combination with in situ hybridization of recorded neurons revealed that hypothalamic NPY-triggered hunger signaling activates GABAergic IRt/PCRt neurons projecting to the rMR [69] (Fig. 5). Furthermore, inactivation of IRt/PCRt neurons eliminates the NPY-induced inhibition of BAT thermogenesis [69] (Fig. 6), indicating the essential role of the IRt/PCRt in the hunger signaling to reduce energy expenditure. These findings raise the model of the neural pathway for metabolic inhibition during hunger: hunger signaling triggered by hypothalamic NPY is transmitted to the IRt/PCRt to activate GABAergic neurons therein, which then inhibit sympathetic premotor neurons in the rMR through their direct projections to suppress BAT thermogenesis. It should be noted that the IRt/PCRt-rMR pathway is unlikely to provide tonic inhibition for the basal control of BAT thermogenesis, because inhibition of IRt/PCRt neurons by itself does not elicit BAT thermogenesis in nonstarved rats under thermoneutral conditions [69].

**Fig. 7:**
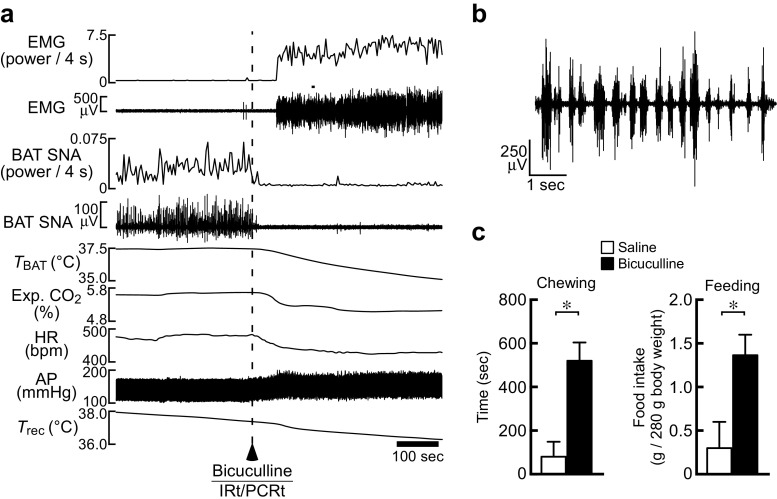
Stimulation of IRt/PCRt neurons promotes mastication and feeding as well as inhibits BAT thermogenesis. **a**, **b** Stimulation of IRt/PCRt neurons with a unilateral nanoinjection of bicuculline in an anesthetized rat elicited mastication and inhibited cooling-evoked BAT thermogenesis and tachycardia (**a**). The masseter electromyogram (EMG) chart (**a**) is expanded in (**b**). **c** Bicuculline injection into the IRt/PCRt in free-moving rats increased chewing time and food intake measured for 1 h after the injection (saline: *n* = 5, bicuculline, *n* = 7). All values are means ± SEM. *P < 0.05. Modified from Nakamura et al. [69] with permission

**Fig. 8:**
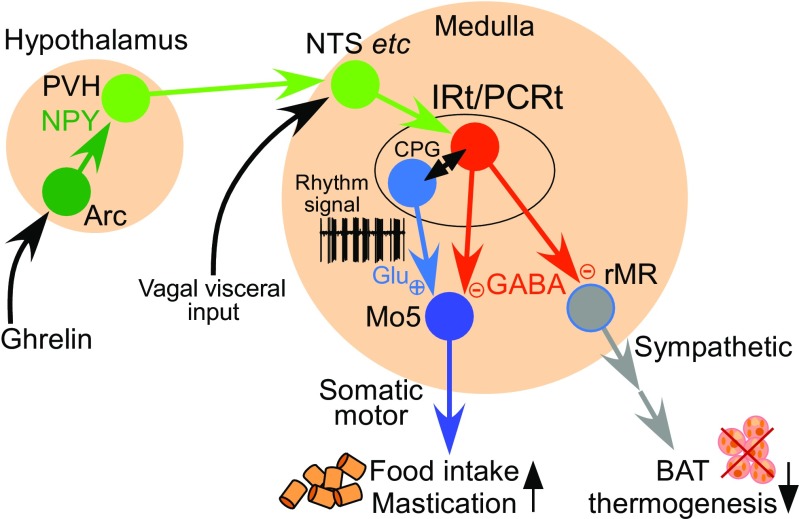
Model of neural circuit that drives hunger responses. NPY-mediated hunger signaling from the hypothalamus is transmitted to the IRt/PCRt through the NTS and/or other brain sites to activate GABAergic neurons (red), which then inhibit the thermogenic sympathetic outflow from the rMR to BAT. These GABAergic IRt/PCRt neurons also facilitate rhythmic premotor signaling to masticatory motoneurons potentially by interacting with glutamatergic neurons constituting the central pattern generator (CPG). For detail, see the main text

Intriguingly, stimulation of IRt/PCRt neurons in rats, which inhibits BAT thermogenesis, also elicits mastication even under anesthesia and increases food intake in free-moving animals, often accompanied by increased saliva secretion [69] (Fig. 7). Furthermore, neural tracing studies revealed that GABAergic IRt/PCRt neurons projecting to the rMR also send their axon collaterals to the motor trigeminal nucleus (Mo5) [69], which harbors masticatory motoneurons [47]. Inactivation of IRt/PCRt neurons reduces mastication and food intake induced by NPY injection into the third ventricle [93]. Therefore, GABAergic neurons in the IRt/PCRt likely act on the two different (sympathetic and somatic) motor systems to drive the NPY-triggered responses to survive hunger and starvation (hunger responses): decreased energy expenditure (thermogenesis) and increased food intake (Fig. 8).

The IRt/PCRt is considered one of the premotor regions that contain the central pattern generator for the rhythmic masticatory muscle movements [48]. Although the mechanism of the pattern generation is unknown, the IRt/PCRt contains both GABAergic and glutamatergic populations of neurons innervating motoneurons in the Mo5 that control jaw muscles [33, 85, 94], and these excitatory and inhibitory premotor inputs from the IRt/PCRt to the Mo5 could be driven for the generation of masticatory motor rhythms [66]. Consistently, some GABAergic IRt/PCRt neurons projecting to the rMR, which also potentially projected to the Mo5, were found to exhibit phasic bursting patterns similar to masticatory rhythm following a central injection of NPY [69]. Therefore, NPY-triggered hunger signaling from the hypothalamus may activate the central pattern generator mechanism in the IRt/PCRt, which then sends rhythmic premotor signals to masticatory Mo5 motoneurons through the glutamatergic and GABAergic projections and simultaneously provides only GABAergic transmission to sympathetic premotor neurons to the rMR to inhibit BAT thermogenesis (Fig. 8). Such masticatory rhythmic premotor signaling could prime the motor system to be “ready to eat,” but the final masticatory motor outflow may be gated by corticomedullary inputs [48, 66], which could be lifted by visual and olfactory sensation of food, to initiate mastication as soon as food is available.

## Hypothalamo-medullary transmission of hunger and satiety signals

The hypothalamic mechanisms by which the brain senses hunger and satiety have been intensively studied. As mentioned above, the ghrelin-NPY signaling pathway is known as one of hunger signaling mechanisms. However, whether the action of NPY on PVH neurons is stimulatory or inhibitory is controversial [46]. Electrophysiological studies have shown that NPY disinhibits PVH neurons by reducing GABA release from presynaptic terminals on PVH neurons [11, 76]. Histochemical analyses have also shown that central administration of NPY induces expression of Fos, a marker for neuronal activation, in the PVH [32]. Consistent with the idea that disinhibition of PVH neurons generates hunger signaling to inhibit BAT thermogenesis, blockade of GABAergic synapses in the PVH with a nanoinjection of a GABA_A_ receptor antagonist or excitation of PVH neurons with a nanoinjection of NMDA inhibits BAT thermogenesis [41]. Similar to the NPY action in the PVH, disinhibition of PVH neurons cannot inhibit BAT thermogenesis induced by antagonizing GABA_A_ receptors in the rMR, indicating BAT sympathoinhibition through GABAergic inhibition of premotor neurons in the rMR [41]. All these findings support the view that disinhibition (or stimulation) of PVH neurons by NPY triggers the hunger signaling that stimulates the IRt/PCRt-rMR GABAergic transmission to inhibit BAT thermogenesis (Fig. 8).

However, NPY can also exert an inhibitory effect on the activity of PVH neurons expressing melanocortin-4 receptors (MC4Rs) through its postsynaptic action [19]. Optogenetic experiments have shown that selective stimulation of AgRP/NPY/GABAergic transmission from the arcuate nucleus to the PVH evokes inhibitory postsynaptic currents in PVH neurons [1]. This optogenetic stimulation increases food intake and this orexigenic effect is reduced by blocking either NPY receptors or GABA_A_ receptors in the PVH [1], suggesting that both NPY and GABA released in the PVH contribute to the trigger of hunger signaling for consummatory responses. It is possible that NPY exerts stimulatory and inhibitory actions on distinct populations of PVH neurons that have different roles in physiological responses to fasting.

The pathway through which the NPY-triggered hunger signals are transmitted from the PVH to the IRt/PCRt has yet to be determined. The existence of few direct projections from the PVH to the IRt/PCRt [82] suggests an indirect pathway(s) mediating the hypothalamomedullary hunger signaling. The nucleus tractus solitarius (NTS) of the medulla oblongata has been proposed as a potential brain site mediating the hunger signaling, as the PVH provides massive projections to this medullary site [18]. The NTS contains neurons that project to GABAergic IRt/PCRt neurons innervating the rMR [69]. Further supporting the idea that the NTS is involved in the neural pathway for hunger responses, stimulation of neurons in the rostral NTS inhibits BAT thermogenesis [69]. Because the PVH contains very few GABAergic neurons [87], the transmission from the PVH to the NTS is considered excitatory, probably glutamatergic. If the PVH-NTS-IRt/PCRt pathway transmits the hunger signals, therefore, the simplest model is that NPY activates excitatory PVH neurons, which then stimulate an NTS-IRt/PCRt excitatory transmission to activate GABAergic IRt/PCRt neurons for the drive of hunger responses (Fig. 8). However, another possibility that NPY-induced inhibition of excitatory PVH neurons triggers the hunger signaling could also stand if a disinhibitory mechanism involving inhibitory neurons, perhaps in the NTS, is postulated. The NPY-triggered PVH-NTS pathway that drives consummatory responses and reduces BAT thermogenic capacity was shown to be inhibited by injections of opioid receptor antagonists into the NTS [29], suggesting an action of endogenous opioids on NTS neurons to modulate the hunger signaling pathway. In light of the large injections of the antagonists, however, these agents might have exerted the effect by acting in other medullary regions around the NTS.

The NTS also receives information on nutritional conditions from visceral organs through the vagus nerve. Increased glucokinase expression in the mouse liver, which is induced by high-fat feeding, leads to decreases in BAT thermogenesis and energy expenditure through the liver–brain vagal afferent [95], suggesting a vagal-mediated mechanism of high-fat diet-induced obesity. The inhibition of BAT thermogenesis by high-fat feeding seems to be mediated by increased glutamatergic vagal inputs to the NTS [42]. Electrical stimulation of the vagus nerve in rats inhibits BAT thermogenesis induced by glutamatergic stimulation of sympathetic premotor neurons in the rMR but does not inhibit that induced by antagonizing GABAergic synapses in the rMR [37], indicating that the vagal-mediated signaling, similar to the hypothalamic NPY-triggered signaling, inhibits BAT sympathetic premotor outflow from the rMR through a GABAergic input to the premotor neurons. This view is consistent with the idea that nutritional vagal visceral signals are integrated with the NPY-triggered hunger signals from the hypothalamus by impinging on NTS neurons before transmitted to the IRt/PCRt (Fig. 8). It should be noted that peripheral ghrelin can act on the vagus nerve to inhibit BAT sympathetic nerve activity [44] in addition to its central action in the arcuate nucleus [27], although ghrelin reduces vagus nerve activity [14].

Another pathway that can be hypothesized to transmit the hypothalamomedullary signaling to the IRt/PCRt is an LPB-mediated pathway. The LPB receives projections from the PVH [18] and provides projections to GABAergic IRt/PCRt neurons innervating the rMR [69]. Excitatory PVH neurons that express MC4Rs have been shown to project to the LPB, but rarely to the NTS, and optogenetic stimulation of their transmission to the LPB attenuates food intake even during hungry conditions [17]. The MC4R is the melanocortin receptor subtype responsible for satiety signaling mediated by α-melanocyte stimulating hormone (α-MSH), which is released from proopiomelanocortin (POMC)-containing neurons in the arcuate nucleus, and deficiency in the MC4R results in hyperphasia and blunted thermogenic responses to increased dietary fat, leading to severe obesity [3, 6, 22]. Among the melanocortin-induced satiety responses, MC4Rs on PVH neurons contribute to attenuation of food intake, but unlikely to increase in energy expenditure [3]. These findings indicate that the PVH-LPB pathway originating from MC4R-expressing PVH neurons transmits satiety signals to stop food intake, likely mediating the satiety responses elicited by leptin’s excitatory action on POMC neurons in the arcuate nucleus [12]. Whether the melanocortin-triggered signaling through the PVH-LPB pathway inhibits GABAergic IRt/PCRt neurons to induce satiety responses has yet to be determined. It should also be noted that melanocortin signaling can increase energy expenditure, potentially BAT thermogenesis, through MC4R-expressing neurons in the DMH [9].

In addition to the postsynaptic inhibitory action of NPY on MC4R-expressing PVH neurons [19], NPY-containing neurons in the arcuate nucleus can inhibit melanocortin signaling by antagonizing MC4Rs in the PVH with the endogenous antagonist, AgRP co-released from their axons to decrease energy expenditure and to increase food intake [11, 83]. In contrast with the rapid stimulation of food intake elicited by NPY and GABA, AgRP induces delayed, chronic feeding [30], suggesting that distinct neural pathways mediate the NPY/GABA-triggered rapid hunger response and the AgRP-triggered slow response. NPY/AgRP/GABA-containing neurons in the arcuate nucleus make synaptic inputs to both MC4R-expressing neurons and non-MC4R neurons in the PVH [17]. Also, NPY can act on diverse groups of PVH neurons that express NPY receptors through its volume transmission even if they do not receive synaptic inputs from NPY neurons. Therefore, NPY may trigger hunger signaling by acting (potentially activating) on a non-MC4R population of PVH neurons to drive the hunger responses through the PVH-NTS-IRt/PCRt pathway. In parallel, inhibitory synaptic actions of NPY and GABA on MC4R-expressing neurons in the PVH with antagonization of MC4R-mediated melanocortin signaling by co-released AgRP likely attenuate satiety signaling from the PVH to the LPB, which might otherwise inhibit IRt/PCRt neurons to reduce food intake. Further studies are required to test this model of hypothalamo-medullary neural pathways transmitting hunger and satiety signals for energy homeostasis.

## Summary and perspective

Studies over the past two decades have made remarkable progress in understanding the central neural circuit mechanisms for body temperature regulation, particularly the control of adaptive thermogenesis in BAT. In the central circuit mechanism controlling BAT thermogenesis, the DMH-rMR-spinal sympathoexcitatory pathway is controlled by tonic GABAergic transmission from the POA, whose activity is altered by body core and cutaneous thermosensory signals. This neural circuit for cold defense also functions to drive BAT thermogenic responses to infection and psychological stress. Therefore, this central circuit mechanism is important not only for thermal homeostasis, but also for the defense of life from various environmental stressors.

Central regulation of BAT thermogenesis dependent on nutritional conditions and food availability is critical for energy homeostasis. Recent studies have revealed that during hunger, a GABAergic group of neurons in the IRt/PCRt of the medulla oblongata takes the control of the two independent (sympathetic and somatic) motor systems to simultaneously command the inhibition of adaptive BAT thermogenesis (energy saving) and the promotion of food intake (energy intake) to survive starvation. The NPY-triggered hunger signaling from the PVH to the IRt/PCRt may be mediated by the NTS, whereas melanocortin-triggered satiety signaling from MC4R-expressing PVH neurons activates LPB neurons, which might inhibit IRt/PCRt neurons to reduce food intake. More physiological investigations are required to determine the mechanisms of the hypothalamomedullary hunger and satiety signal transmission. In addition to the aforementioned humoral and neural (vagal) transmission of hunger and satiety signals from the periphery to the brain, central sensing of fatty acids and glucose levels [74, 75] also affects the central regulation of BAT thermogenesis and energy homeostasis [15, 36, 43]. Further studies to elucidate the whole circuit mechanism regulating energy and thermal homeostasis will pave the way to novel therapeutic strategies for symptoms related to energy imbalance, such as obesity and anorexia nervosa.

## References

[1] Atasoy D, Betley JN, Su HH, Sternson SM (2012). Deconstruction of a neural circuit for hunger. Nature.

[2] Bachman ES, Dhillon H, Zhang CY, Cinti S, Bianco AC, Kobilka BK, Lowell BB (2002). βAR signaling required for diet-induced thermogenesis and obesity resistance. Science.

[3] Balthasar N, Dalgaard LT, Lee CE, Yu J, Funahashi H, Williams T, Ferreira M, Tang V, McGovern RA, Kenny CD, Christiansen LM, Edelstein E, Choi B, Boss O, Aschkenasi C, Zhang CY, Mountjoy K, Kishi T, Elmquist JK, Lowell BB (2005). Divergence of melanocortin pathways in the control of food intake and energy expenditure. Cell.

[4] Bartelt A, Bruns OT, Reimer R, Hohenberg H, Ittrich H, Peldschus K, Kaul MG, Tromsdorf UI, Weller H, Waurisch C, Eychmüller A, Gordts PLSM, Rinninger F, Bruegelmann K, Freund B, Nielsen P, Merkel M, Heeren J (2011). Brown adipose tissue activity controls triglyceride clearance. Nat Med.

[5] Boulant JA, Hardy JD (1974). The effect of spinal and skin temperatures on the firing rate and thermosensitivity of preoptic neurones. J Physiol.

[6] Butler AA, Marks DL, Fan W, Kuhn CM, Bartolome M, Cone RD (2001). Melanocortin-4 receptor is required for acute homeostatic responses to increased dietary fat. Nat Neurosci.

[7] Cannon B, Nedergaard J (2004). Brown adipose tissue: function and physiological significance. Physiol Rev.

[8] Cao WH, Fan W, Morrison SF (2004). Medullary pathways mediating specific sympathetic responses to activation of dorsomedial hypothalamus. Neuroscience.

[9] Chen M, Shrestha YB, Podyma B, Cui Z, Naglieri B, Sun H, Ho T, Wilson EA, Li YQ, Gavrilova O, Weinstein LS (2017). G_s_α deficiency in the dorsomedial hypothalamus underlies obesity associated with G_s_α mutations. J Clin Invest.

[10] Chen XM, Hosono T, Yoda T, Fukuda Y, Kanosue K (1998). Efferent projection from the preoptic area for the control of non-shivering thermogenesis in rats. J Physiol.

[11] Cowley MA, Pronchuk N, Fan W, Dinulescu DM, Colmers WF, Cone RD (1999). Integration of NPY, AGRP, and melanocortin signals in the hypothalamic paraventricular nucleus: evidence of a cellular basis for the adipostat. Neuron.

[12] Cowley MA, Smart JL, Rubinstein M, Cerdán MG, Diano S, Horvath TL, Cone RD, Low MJ (2001). Leptin activates anorexigenic POMC neurons through a neural network in the arcuate nucleus. Nature.

[13] Cypess AM, Lehman S, Williams G, Tal I, Rodman D, Goldfine AB, Kuo FC, Palmer EL, Tseng YH, Doria A, Kolodny GM, Kahn CR (2009). Identification and importance of brown adipose tissue in adult humans. N Engl J Med.

[14] Date Y, Murakami N, Toshinai K, Matsukura S, Niijima A, Matsuo H, Kangawa K, Nakazato M (2002). The role of the gastric afferent vagal nerve in ghrelin-induced feeding and growth hormone secretion in rats. Gastroenterology.

[15] Egawa M, Yoshimatsu H, Bray GA (1989). Effects of 2-deoxy-D-glucose on sympathetic nerve activity to interscapular brown adipose tissue. Am J Phys.

[16] Egawa M, Yoshimatsu H, Bray GA (1991). Neuropeptide Y suppresses sympathetic activity to interscapular brown adipose tissue in rats. Am J Phys.

[17] Garfield AS, Li C, Madara JC, Shah BP, Webber E, Steger JS, Campbell JN, Gavrilova O, Lee CE, Olson DP, Elmquist JK, Tannous BA, Krashes MJ, Lowell BB (2015). A neural basis for melanocortin-4 receptor-regulated appetite. Nat Neurosci.

[18] Geerling JC, Shin JW, Chimenti PC, Loewy AD (2010). Paraventricular hypothalamic nucleus: axonal projections to the brainstem. J Comp Neurol.

[19] Ghamari-Langroudi M, Srisai D, Cone RD (2011). Multinodal regulation of the arcuate/paraventricular nucleus circuit by leptin. Proc Natl Acad Sci U S A.

[20] Himms-Hagen J, Cui J, Danforth E, Taatjes DJ, Lang SS, Waters BL, Claus TH (1994). Effect of CL-316,243, a thermogenic β_3_-agonist, on energy balance and brown and white adipose tissues in rats. Am J Phys.

[21] Hosoya Y, Ito R, Kohno K (1987). The topographical organization of neurons in the dorsal hypothalamic area that project to the spinal cord or to the nucleus raphé pallidus in the rat. Exp Brain Res.

[22] Huszar D, Lynch CA, Fairchild-Huntress V, Dunmore JH, Fang Q, Berkemeier LR, Gu W, Kesterson RA, Boston BA, Cone RD, Smith FJ, Campfield LA, Burn P, Lee F (1997). Targeted disruption of the melanocortin-4 receptor results in obesity in mice. Cell.

[23] Imai-Matsumura K, Matsumura K, Nakayama T (1984). Involvement of ventromedial hypothalamus in brown adipose tissue thermogenesis induced by preoptic cooling in rats. Jpn J Physiol.

[24] Kalra SP, Dube MG, Sahu A, Phelps CP, Kalra PS (1991). Neuropeptide Y secretion increases in the paraventricular nucleus in association with increased appetite for food. Proc Natl Acad Sci U S A.

[25] Kanosue K, Crawshaw LI, Nagashima K, Yoda T (2010). Concepts to utilize in describing thermoregulation and neurophysiological evidence for how the system works. Eur J Appl Physiol.

[26] Kataoka N, Hioki H, Kaneko T, Nakamura K (2014). Psychological stress activates a dorsomedial hypothalamus-medullary raphe circuit driving brown adipose tissue thermogenesis and hyperthermia. Cell Metab.

[27] Kohno D, Gao HZ, Muroya S, Kikuyama S, Yada T (2003) Ghrelin directly interacts with neuropeptide-Y-containing neurons in the rat arcuate nucleus: Ca^2+^ signaling via protein kinase a and N-type channel-dependent mechanisms and cross-talk with leptin and orexin. Diabetes 52(4):948–956. 10.2337/diabetes.52.4.948 10.2337/diabetes.52.4.948 12663466

[28] Kojima M, Kangawa K (2005). Ghrelin: structure and function. Physiol Rev.

[29] Kotz CM, Grace MK, Briggs J, Levine AS, Billington CJ (1995). Effects of opioid antagonists naloxone and naltrexone on neuropeptide Y-induced feeding and brown fat thermogenesis in the rat. Neural site of action. J Clin Invest.

[30] Krashes MJ, Shah BP, Koda S, Lowell BB (2013). Rapid versus delayed stimulation of feeding by the endogenously released AgRP neuron mediators GABA, NPY, and AgRP. Cell Metab.

[31] Lazarus M, Yoshida K, Coppari R, Bass CE, Mochizuki T, Lowell BB, Saper CB (2007). EP3 prostaglandin receptors in the median preoptic nucleus are critical for fever responses. Nat Neurosci.

[32] Li BH, Xu B, Rowland NE, Kalra SP (1994). C-*fos* expression in the rat brain following central administration of neuropeptide Y and effects of food consumption. Brain Res.

[33] Li YQ, Takada M, Kaneko T, Mizuno N (1996). GABAergic and glycinergic neurons projecting to the trigeminal motor nucleus: a double labeling study in the rat. J Comp Neurol.

[34] Lkhagvasuren B, Nakamura Y, Oka T, Sudo N, Nakamura K (2011). Social defeat stress induces hyperthermia through activation of thermoregulatory sympathetic premotor neurons in the medullary raphe region. Eur J Neurosci.

[35] Lowell BB, S-Susulic V, Hamann A, Lawitts JA, Himms-Hagen J, Boyer BB, Kozak LP, Flier JS (1993). Development of obesity in transgenic mice after genetic ablation of brown adipose tissue. Nature.

[36] Madden CJ (2012). Glucoprivation in the ventrolateral medulla decreases brown adipose tissue sympathetic nerve activity by decreasing the activity of neurons in raphe pallidus. Am J Physiol Regul Integr Comp Physiol.

[37] Madden CJ, da Conceição EPS, Morrison SF (2017). Vagal afferent activation decreases brown adipose tissue (BAT) sympathetic nerve activity and BAT thermogenesis. Temperature.

[38] Madden CJ, Morrison SF (2003). Excitatory amino acid receptor activation in the raphe pallidus area mediates prostaglandin-evoked thermogenesis. Neuroscience.

[39] Madden CJ, Morrison SF (2004). Excitatory amino acid receptors in the dorsomedial hypothalamus mediate prostaglandin-evoked thermogenesis in brown adipose tissue. Am J Physiol Regul Integr Comp Physiol.

[40] Madden CJ, Morrison SF (2006). Serotonin potentiates sympathetic responses evoked by spinal NMDA. J Physiol.

[41] Madden CJ, Morrison SF (2009). Neurons in the paraventricular nucleus of the hypothalamus inhibit sympathetic outflow to brown adipose tissue. Am J Physiol Regul Integr Comp Physiol.

[42] Madden CJ, Morrison SF (2016). A high-fat diet impairs cooling-evoked brown adipose tissue activation via a vagal afferent mechanism. Am J Physiol Endocrinol Metab.

[43] Magnan C, Levin BE, Luquet S (2015) Brain lipid sensing and the neural control of energy balance. Mol Cell Endocrinol 418(1):3–8. 10.1016/j.mce.2015.09.019 10.1016/j.mce.2015.09.019 26415589

[44] Mano-Otagiri A, Ohata H, Iwasaki-Sekino A, Nemoto T, Shibasaki T (2009). Ghrelin suppresses noradrenaline release in the brown adipose tissue of rats. J Endocrinol.

[45] Matsumura K, Cao C, Ozaki M, Morii H, Nakadate K, Watanabe Y (1998). Brain endothelial cells express cyclooxygenase-2 during lipopolysaccharide-induced fever: light and electron microscopic immunocytochemical studies. J Neurosci.

[46] Mercer RE, Chee MJ, Colmers WF (2011). The role of NPY in hypothalamic mediated food intake. Front Neuroendocrinol.

[47] Mizuno N, Konishi A, Sato M (1975). Localization of masticatory motoneurons in the cat and rat by means of retrograde axonal transport of horseradish peroxidase. J Comp Neurol.

[48] Moore JD, Kleinfeld D, Wang F (2014). How the brainstem controls orofacial behaviors comprised of rhythmic actions. Trends Neurosci.

[49] Morrison SF (1999). RVLM and raphe differentially regulate sympathetic outflows to splanchnic and brown adipose tissue. Am J Physiol Regul Integr Comp Physiol.

[50] Morrison SF (2003). Raphe pallidus neurons mediate prostaglandin E_2_-evoked increases in brown adipose tissue thermogenesis. Neuroscience.

[51] Morrison SF, Nakamura K (2011) Central neural pathways for thermoregulation. Front Biosci 16:74–104. 10.2741/3677 10.2741/3677 PMC3051412 21196160

[52] Morrison SF, Sved AF, Passerin AM (1999). GABA-mediated inhibition of raphe pallidus neurons regulates sympathetic outflow to brown adipose tissue. Am J Physiol Regul Integr Comp Physiol.

[53] Muzzin P, Revelli JP, Kuhne F, Gocayne JD, McCombie WR, Venter JC, Giacobino JP, Fraser CM (1991). An adipose tissue-specific β-adrenergic receptor. Molecular cloning and down-regulation in obesity. J Biol Chem.

[54] Nakamura K (2011). Central circuitries for body temperature regulation and fever. Am J Physiol Regul Integr Comp Physiol.

[55] Nakamura K, Kaneko T, Yamashita Y, Hasegawa H, Katoh H, Ichikawa A, Negishi M (1999). Immunocytochemical localization of prostaglandin EP3 receptor in the rat hypothalamus. Neurosci Lett.

[56] Nakamura K, Kaneko T, Yamashita Y, Hasegawa H, Katoh H, Negishi M (2000). Immunohistochemical localization of prostaglandin EP3 receptor in the rat nervous system. J Comp Neurol.

[57] Nakamura K, Matsumura K, Hübschle T, Nakamura Y, Hioki H, Fujiyama F, Boldogköi Z, König M, Thiel H-J, Gerstberger R, Kobayashi S, Kaneko T (2004). Identification of sympathetic premotor neurons in medullary raphe regions mediating fever and other thermoregulatory functions. J Neurosci.

[58] Nakamura K, Matsumura K, Kaneko T, Kobayashi S, Katoh H, Negishi M (2002) The rostral raphe pallidus nucleus mediates pyrogenic transmission from the preoptic area. J Neurosci 22(11):4600–4610 10.1523/JNEUROSCI.22-11-04600.2002 PMC6758794 12040067

[59] Nakamura K, Matsumura K, Kobayashi S, Kaneko T (2005) Sympathetic premotor neurons mediating thermoregulatory functions. Neurosci Res 51(1):1–8. 10.1016/j.neures.2004.09.007 10.1016/j.neures.2004.09.007 15596234

[60] Nakamura K, Morrison SF (2007). Central efferent pathways mediating skin cooling-evoked sympathetic thermogenesis in brown adipose tissue. Am J Physiol Regul Integr Comp Physiol.

[61] Nakamura K, Morrison SF (2008). A thermosensory pathway that controls body temperature. Nat Neurosci.

[62] Nakamura K, Morrison SF (2008). Preoptic mechanism for cold-defensive responses to skin cooling. J Physiol.

[63] Nakamura K, Morrison SF (2010). A thermosensory pathway mediating heat-defense responses. Proc Natl Acad Sci U S A.

[64] Nakamura K, Morrison SF (2011). Central efferent pathways for cold-defensive and febrile shivering. J Physiol.

[65] Nakamura K, Wu SX, Fujiyama F, Okamoto K, Hioki H, Kaneko T (2004). Independent inputs by VGLUT2- and VGLUT3-positive glutamatergic terminals onto rat sympathetic preganglionic neurons. Neuroreport.

[66] Nakamura Y, Katakura N (1995). Generation of masticatory rhythm in the brainstem. Neurosci Res.

[67] Nakamura Y, Nakamura K, Matsumura K, Kobayashi S, Kaneko T, Morrison SF (2005). Direct pyrogenic input from prostaglandin EP3 receptor-expressing preoptic neurons to the dorsomedial hypothalamus. Eur J Neurosci.

[68] Nakamura Y, Nakamura K, Morrison SF (2009). Different populations of prostaglandin EP3 receptor-expressing preoptic neurons project to two fever-mediating sympathoexcitatory brain regions. Neuroscience.

[69] Nakamura Y, Yanagawa Y, Morrison SF, Nakamura K (2017). Medullary reticular neurons mediate neuropeptide Y-induced metabolic inhibition and mastication. Cell Metab.

[70] Nakayama T, Eisenman JS, Hardy JD (1961). Single unit activity of anterior hypothalamus during local heating. Science.

[71] Nakayama T, Hammel HT, Hardy JD, Eisenman JS (1963) Thermal stimulation of electrical activity of single units of the preoptic region. Am J Physiol 204(6):1122–1126

[72] Narumiya S, Sugimoto Y, Ushikubi F (1999). Prostanoid receptors: structures, properties, and functions. Physiol Rev.

[73] Nason MW, Mason P (2004). Modulation of sympathetic and somatomotor function by the ventromedial medulla. J Neurophysiol.

[74] Oomura Y, Nakamura T, Sugimori M, Yamada Y (1975). Effect of free fatty acid on the rat lateral hypothalamic neurons. Physiol Behav.

[75] Oomura Y, Ono T, Ooyama H, Wayner MJ (1969). Glucose and osmosensitive neurones of the rat hypothalamus. Nature.

[76] Pronchuk N, Beck-Sickinger AG, Colmers WF (2002). Multiple NPY receptors inhibit GABA_A_ synaptic responses of rat medial parvocellular effector neurons in the hypothalamic paraventricular nucleus. Endocrinology.

[77] Rathner JA, Madden CJ, Morrison SF (2008). Central pathway for spontaneous and prostaglandin E_2_-evoked cutaneous vasoconstriction. Am J Physiol Regul Integr Comp Physiol.

[78] Saito M, Okamatsu-Ogura Y, Matsushita M, Watanabe K, Yoneshiro T, Nio-Kobayashi J, Iwanaga T, Miyagawa M, Kameya T, Nakada K, Kawai Y, Tsujisaki M (2009). High incidence of metabolically active brown adipose tissue in healthy adult humans: effects of cold exposure and adiposity. Diabetes.

[79] Sakurada S, Shido O, Sugimoto N, Hiratsuka Y, Yoda T, Kanosue K (2000). Autonomic and behavioural thermoregulation in starved rats. J Physiol.

[80] Samuels BC, Zaretsky DV, DiMicco JA (2004). Dorsomedial hypothalamic sites where disinhibition evokes tachycardia correlate with location of raphe-projecting neurons. Am J Physiol Regul Integr Comp Physiol.

[81] Sarkar S, Zaretskaia MV, Zaretsky DV, Moreno M, DiMicco JA (2007). Stress- and lipopolysaccharide-induced c-fos expression and nNOS in hypothalamic neurons projecting to medullary raphe in rats: a triple immunofluorescent labeling study. Eur J Neurosci.

[82] Shammah-Lagnado SJ, Costa MSMO, Ricardo JA (1992). Afferent connections of the parvocellular reticular formation: a horseradish peroxidase study in the rat. Neuroscience.

[83] Sohn JW, Elmquist JK, Williams KW (2013). Neuronal circuits that regulate feeding behavior and metabolism. Trends Neurosci.

[84] Song K, Wang H, Kamm GB, Pohle J, Reis FC, Heppenstall P, Wende H, Siemens J (2016). The TRPM2 channel is a hypothalamic heat sensor that limits fever and can drive hypothermia. Science.

[85] Stanek E, Cheng S, Takatoh J, Han BX, Wang F (2014). Monosynaptic premotor circuit tracing reveals neural substrates for oro-motor coordination. Elife.

[86] Stanley BG, Leibowitz SF (1985). Neuropeptide Y injected in the paraventricular hypothalamus: a powerful stimulant of feeding behavior. Proc Natl Acad Sci U S A.

[87] Stocker SD, Simmons JR, Stornetta RL, Toney GM, Guyenet PG (2006). Water deprivation activates a glutamatergic projection from the hypothalamic paraventricular nucleus to the rostral ventrolateral medulla. J Comp Neurol.

[88] Stornetta RL, Rosin DL, Simmons JR, McQuiston TJ, Vujovic N, Weston MC, Guyenet PG (2005). Coexpression of vesicular glutamate transporter-3 and gamma-aminobutyric acidergic markers in rat rostral medullary raphe and intermediolateral cell column. J Comp Neurol.

[89] Susulic VS, Frederich RC, Lawitts J, Tozzo E, Kahn BB, Harper ME, Himms-Hagen J, Flier JS, Lowell BB (1995). Targeted disruption of the β_3_-adrenergic receptor gene. J Biol Chem.

[90] Székely M, Pétervári E, Pákai E, Hummel Z, Szelényi Z (2005). Acute, subacute and chronic effects of central neuropeptide Y on energy balance in rats. Neuropeptides.

[91] Tabarean IV, Conti B, Behrens M, Korn H, Bartfai T (2005). Electrophysiological properties and thermosensitivity of mouse preoptic and anterior hypothalamic neurons in culture. Neuroscience.

[92] Tan CL, Cooke EK, Leib DE, Lin YC, Daly GE, Zimmerman CA, Knight ZA (2016). Warm-sensitive neurons that control body temperature. Cell.

[93] Travers JB, Herman K, Travers SP (2010). Suppression of third ventricular NPY-elicited feeding following medullary reticular formation infusions of muscimol. Behav Neurosci.

[94] Travers JB, Yoo JE, Chandran R, Herman K, Travers SP (2005). Neurotransmitter phenotypes of intermediate zone reticular formation projections to the motor trigeminal and hypoglossal nuclei in the rat. J Comp Neurol.

[95] Tsukita S, Yamada T, Uno K, Takahashi K, Kaneko K, Ishigaki Y, Imai J, Hasegawa Y, Sawada S, Ishihara H, Oka Y, Katagiri H (2012). Hepatic glucokinase modulates obesity predisposition by regulating BAT thermogenesis via neural signals. Cell Metab.

[96] van Marken Lichtenbelt WD, Vanhommerig JW, Smulders NM, Drossaerts JMAFL, Kemerink GJ, Bouvy ND, Schrauwen P, Teule GJJ (2009). Cold-activated brown adipose tissue in healthy men. N Engl J Med.

[97] Virtanen KA, Lidell ME, Orava J, Heglind M, Westergren R, Niemi T, Taittonen M, Laine J, Savisto NJ, Enerbäck S, Nuutila P (2009). Functional brown adipose tissue in healthy adults. N Engl J Med.

[98] Walker HC, Romsos DR (1993) Similar effects of NPY on energy metabolism and on plasma insulin in adrenalectomized ob/ob and lean mice. Am J Physiol 264:E226–E230 10.1152/ajpendo.1993.264.2.E226 8447389

[99] Wilhelms DB, Kirilov M, Mirrasekhian E, Eskilsson A, Kugelberg UÖ, Klar C, Ridder DA, Herschman HR, Schwaninger M, Blomqvist A, Engblom D (2014). Deletion of prostaglandin E_2_ synthesizing enzymes in brain endothelial cells attenuates inflammatory fever. J Neurosci.

[100] Yamagata K, Matsumura K, Inoue W, Shiraki T, Suzuki K, Yasuda S, Sugiura H, Cao C, Watanabe Y, Kobayashi S (2001). Coexpression of microsomal-type prostaglandin E synthase with cyclooxygenase-2 in brain endothelial cells of rats during endotoxin-induced fever. J Neurosci.

[101] Yoshida K, Li X, Cano G, Lazarus M, Saper CB (2009). Parallel preoptic pathways for thermoregulation. J Neurosci.

[102] Yoshida K, Nakamura K, Matsumura K, Kanosue K, König M, Thiel HJ, Boldogköi Z, Toth I, Roth J, Gerstberger R, Hübschle T (2003). Neurons of the rat preoptic area and the raphe pallidus nucleus innervating the brown adipose tissue express the prostaglandin E receptor subtype EP3. Eur J Neurosci.

[103] Young JB, Saville E, Rothwell NJ, Stock MJ, Landsberg L (1982). Effect of diet and cold exposure on norepinephrine turnover in brown adipose tissue of the rat. J Clin Invest.

[104] Yu S, Qualls-Creekmore E, Rezai-Zadeh K, Jiang Y, Berthoud HR, Morrison CD, Derbenev AV, Zsombok A, Münzberg H (2016). Glutamatergic preoptic area neurons that express leptin receptors drive temperature-dependent body weight homeostasis. J Neurosci.

[105] Zaretskaia MV, Zaretsky DV, DiMicco JA (2003). Role of the dorsomedial hypothalamus in thermogenesis and tachycardia caused by microinjection of prostaglandin E2 into the preoptic area in anesthetized rats. Neurosci Lett.

[106] Zaretskaia MV, Zaretsky DV, Shekhar A, DiMicco JA (2002). Chemical stimulation of the dorsomedial hypothalamus evokes non-shivering thermogenesis in anesthetized rats. Brain Res.

[107] Zhang LN, Mitchell SE, Hambly C, Morgan DG, Clapham JC, Speakman JR (2012). Physiological and behavioral responses to intermittent starvation in C57BL/6J mice. Physiol Behav.

